# Mental wellbeing among Danish employees during the COVID-19 pandemic: results from a longitudinal study on the role of industry and working environment

**DOI:** 10.1093/eurpub/ckac150

**Published:** 2022-10-13

**Authors:** Maj Britt Dahl Nielsen, Ola Ekholm, Sanne Pagh Møller, Annette Kjær Ersbøll, Ziggi Ivan Santini, Morten Klöcker Grønbæk, Lau Caspar Thygesen

**Affiliations:** National Institute of Public Health, University of Southern Denmark, Copenhagen, Denmark; National Institute of Public Health, University of Southern Denmark, Copenhagen, Denmark; National Institute of Public Health, University of Southern Denmark, Copenhagen, Denmark; National Institute of Public Health, University of Southern Denmark, Copenhagen, Denmark; National Institute of Public Health, University of Southern Denmark, Copenhagen, Denmark; National Institute of Public Health, University of Southern Denmark, Copenhagen, Denmark; National Institute of Public Health, University of Southern Denmark, Copenhagen, Denmark

## Abstract

**Background:**

The COVID-19 pandemic has had a profound impact on working life. Previous studies have primarily focused on the mental health and wellbeing of healthcare workers and are mostly based on cross-sectional data from non-representative samples. The aim of this study was to investigate mental wellbeing trajectories among employees from different industries, and to longitudinally identify factors that affect mental wellbeing during the COVID-19 pandemic, including job insecurity, fear of COVID-19, working from home or being discharged with wage compensation and management quality.

**Methods:**

Baseline data were obtained from the Danish Health and Wellbeing Survey in 2019 (September–December), with follow-up in September–November 2020. We included 1995 respondents, who completed the questionnaire in both waves and were employed in 2020 and measured mental wellbeing using the Short Warwick–Edinburgh Mental Well-Being Scale.

**Results:**

Mental wellbeing declined among employees in all industries. Employees working from home and employees unsatisfied with management experienced a greater decline in mental wellbeing. We found no differences in mental wellbeing trajectories in relation to fear of infecting others or contracting COVID-19, job insecurity and being discharged with wage compensation.

**Conclusions:**

Mental wellbeing declined among employees in all industries with no difference between industries. Employees working from home may have been particularly vulnerable, and the analyses show that managers play a key role in mitigating the negative consequences of the pandemic by ensuring adequate information and involvement of employees.

## Introduction

In January 2020, the World Health Organization^1^ declared COVID-19 a global emergency, and governments responded with national lockdowns, travel bans, border shutdowns, quarantines and social distancing measures.^2^ From one day to another, COVID-19 dramatically changed everyday life and working arrangements. Working from home became the customary way of working for many employees with limited or no prior experience.^3^ In the European Union (EU), half of all employees worked from home in July 2020. While working from home was particularly widespread among white collar workers, many essential and frontline workers did not have that same opportunity. About 44% of employees in the EU believed they were at risk of contracting COVID-19 at their workplace, and these concerns were particularly prevalent in jobs requiring contact with other people, such as health care, hospitality, transport and education.^3^ Healthcare and frontline workers play a vital role in our response to COVID-19, and research shows that they are likely to experience mental disorders and reduced mental health [including depression, anxiety, post-traumatic stress disorder (PTSD), sleep problems, burnout and distress] during epidemics.^4^^,^^5^ In an epidemic, the number of patients increases markedly, placing a tremendous amount of pressure on staff.^6^ Moreover, lack of personal protective equipment and training can exacerbate the negative mental health impacts associated with the COVID-19 pandemic.^4^

COVID-19 not only constitutes a health crisis, but it is also a social and economic crisis. Especially, the tourism and hospitality sector have experienced major financial challenges worldwide.^2^ Job insecurity is a known risk factor for depression and anxiety,^7^ and employees from the hospitality sector may therefore also be at high risk of experiencing mental health problems during the pandemic. Moreover, previous research show that major organizational changes can have negative effects on employee’s wellbeing, job satisfaction and mental health.^8^^,^^9^ To cope, employees will attempt to gather information to construe meaning of changes. Managers can help employees make sense and reduce uncertainty by providing timely and adequate information and involving employees in change processes.^9^

To date, most studies focus on healthcare workers, and research comparing trends across different industries are scarce.^4^ While numerous studies have shown relatively high rates of mental health problems (including depression, anxiety, PTSD and stress) during the pandemic, most studies are cross-sectional with no pre-pandemic measures and based on convenience samples.^10–13^ The urgent need to produce critical information may have been on the expense of high-quality research posing a threat to the validity, generalizability and replicability of results.^14^ In this study, we measured mental wellbeing in a representative sample of the Danish population just before the pandemic in 2019 and again in 2020. The aim of this study is to investigate mental health trajectories among employees from different industries, and to identify factors that positively or negatively affect mental wellbeing. Knowledge about high-risk groups is important for targeting public mental health efforts to those who are most vulnerable.

Drawing on Conservation of Resources (COR) theory, we hypothesize that COVID-19 may have detrimental effects on employee’s wellbeing by introducing a significant resource loss, i.e. due to job insecurity, higher uncertainty and loss of socialization.^15^ More specifically, we hypothesized that employees from health- and social care and the hospitality industry were more likely to experience a decrease in mental wellbeing compared to other employees. Furthermore, we hypothesized that fear of becoming infected with COVID-19 and job insecurity constitute a threat of a loss of important resources and therefore a decline in mental wellbeing. Finally, we hypothesize that receiving adequate information and high management quality is associated with better mental wellbeing, because social support (emotional and/or practical) can mitigate the negative consequence of resource loss.^16^

## Methods

### Design and data collection

We used data from the Danish Health and Wellbeing Survey, which we distributed to a representative sample of the Danish population in September–December 2019 and again in September–November 2020. Residents in Denmark have a personal identification number, which is used throughout administrative registers and stored in the Civil Registration System.^17^ From the Civil Registration System, we randomly selected and invited 14 000 persons aged 15 years or more to the 2019 survey. In total, 6629 persons (47%) completed the survey. We re-invited all who were still alive and living in Denmark in mid-August 2020 (excluding persons with specific reasons for non-response in 2019, e.g. hard refusers, persons with severe cognitive impairment and persons not understanding Danish). Thus, 13 474 persons were re-invited in 2020.^18^ In all, 6712 individuals completed the self-administered questionnaire in 2020, out of which 5000 had also completed the questionnaire in 2019.

We included respondents who completed the Short Warwick–Edinburgh Mental Well-Being Scale (SWEMWBS) in 2019 and 2020 (*n* = 4234) and who responded that they were employed in 2020 (*n* = 2253). Finally, we excluded 258 responders with missing data on industry. Thus, the final sample consisted of 1995 responders employed in industries from the primary and secondary sector (*n* = 345), trade and transport (*n* = 299), hospitality (*n* = 133), other liberal professions (*n* = 349), public administration and education (*n* = 410) and health- and social care (*n* = 459).

Participation in the surveys was voluntary, which was informed to the invitees. In Denmark, register and questionnaire studies do not require approval by committees on biomedical research ethics according to Danish legislation. The surveys in 2019 and 2020 were approved by SDU Research & Innovation Organization (RIO). Since 2016, RIO examines and approves all scientific and statistical projects at the University of Southern Denmark according to the Danish Data Protection Regulation.

The 2019 survey covered a range of topics including: health (e.g. self-rated health, chronic conditions, mental health and accidents), health determinants (e.g. smoking, alcohol consumption and physical activity), healthcare utilization (e.g. use of different types of health-care services) and social- and demographic characteristics (e.g. marital status and labour market participation). The 2020 survey also covered aspects related to the COVID-19 pandemic. The details of the design and data collection have been reported elsewhere.^18^ In the analyses in this article, we included respondents who reported that they were employed in September 2020.

### Setting

The data were collected September–December in 2019 well before the COVID-19 pandemic reached Denmark and again in September–November 2020. Between these timepoints, Denmark experienced its first COVID-19 wave. In March, the Government closed all schools, day care facilities and cultural institutions. All non-essential public servants were sent home, private companies’ employees were encouraged to work from home, the border was closed and travel restrictions implemented. Restaurants, cafes, shopping centres, fitness centres, and leisure-activities, libraries, museums and shops were closed by 18 March 2020. The Danish Government also passed several relief packages for private businesses in Denmark, including a wage compensation scheme to enable companies to retain their employees. This scheme allowed the state to reimburse employers for 75% of employee’s salary (up to max. 4034 EUR per month) for employees who were sent home, while retaining jobs with full salary. A gradual reopening started in April 2020, and in June–August 2020 only minor restrictions were still in place, although the general request to work from home was not lifted. By August 2020 the number of COVID-19 cases increased, and the use of face masks in public indoor areas became mandatory. During September and October 2020, the Government reinstated travel restrictions and the number of persons to engage with.^19^

### Measures

#### Mental wellbeing

We measured mental wellbeing using SWEMWBS. Both Danish translations of the scales have been validated in Denmark.^20^ SWEMWBS consists of seven positively worded questions pertaining to mental wellbeing experienced within the past 14 days: (i) I have been feeling optimistic about the future, (ii) I have been feeling useful, (iii) I have been feeling relaxed, (iv) I have been dealing with problems well, (v) I have been thinking clearly, (vi) I have been feeling close to other people and (vii) I have been able to make up my own mind about things. Response options were none of the time (1 point); rarely (2 points); some of the time (3 points); often (4 points); and all of the time (5 points). We included SWEMWBS as a continuous variable. Summing item scores leads to a score between 7 and 35, the higher the score, the higher mental wellbeing. The final scores were then transformed to enhance scaling properties (available online).^20^

#### Industry

We retrieved data on industry from the Integrated Database for Labour Market Research in September 2020 and divided industries into six categories using the codes of the Danish version of the EU’s nomenclature (NACE, Statistical classification of economic activities in the European Community) from Statistics Denmark. We categorized industries into six groups: (i) industries from the primary and secondary sector and transport (agriculture, forestry and fishing agriculture, manufacturing, mining and quarrying, and utility services, and construction); (ii) trade (wholesale and retail) and transport; (iii) hospitality (including arts, entertainment and other services, accommodation and food service activities, i.e. hotels and restaurants); (iv) other liberal professions (including information and communication, financial and insurance, real estate and other business services, i.e. knowledge-based services); (v) public administration and education; and (vi) health and social care (i.e. hospitals, medical and dental practices and residential care).

#### Working from home and other working arrangements during the lockdown

We asked the respondents about their work situation in the 12 weeks from mid-March to June 2020 (marking the beginning of the national lockdown in Denmark). The respondents could choose between different responses, including: ‘I did not have a job’, ‘I was sent home from work with wage compensation’, ‘I was sent home from work without wage compensation’, ‘I have been coming into work physically’ and ‘I have been working from home/remotely’*.* Subsequently, respondents were asked to specify the number of weeks of each working arrangement.

#### Fear of COVID-19 and job insecurity

To measure fear of COVID-19, we asked the respondents to what degree they were worried about contracting COVID-19 themselves and to infect others during the lockdown (response categories; ‘to a very high degree’, ‘to a high degree’, ‘somewhat’, ‘to a low degree’ and ‘to a very low degree’). We measured job insecurity with a single item: ‘To what degree are you worried about becoming unemployed?’ using the same response categories as in the question regarding fear of COVID-19. Responses were coded into high (to a very high and high degree) and low (somewhat, to a low and a very low degree) degree of fear of contracting COVID-19 and infecting others and high and low job insecurity.

#### Management quality during COVID-19

First, we asked respondents if they had experienced changes in their workplace during the lockdown and the reopening in Denmark. Next, to those respondents who replied in the affirmative, we asked three questions about the management quality, adapted from the Danish Psychosocial Work Environment Questionnaire^21^: ‘Has management adequately informed about changes at work?’, ‘Have employees been adequately involved in the changes?’ and ‘In general, are you satisfied with the way management has managed the changes?’. We used the same response categories as in the questions about job insecurity and fear of COVID-19.

### Statistical analyses

Descriptive statistics were used to describe participants by industry. Proportions, means and medians were calculated. To examine changes in SWEMWBS from 2019 to 2020, linear repeated measurements regression models were conducted. The main interest was on whether mean SWEMWBS in 2019 and 2020 were different in different industries. This was evaluated by an overall type-3 test of interaction between time (2019 or 2020) and industries. All analyses were adjusted for sex and age. Age was included as a continuous variable, and as second- and third-degree polynomials. To take account of the correlation due to the repeated measurements for the same person, we estimated generalized estimation equations linear models assuming an exchangeable correlation matrix. Similar analyses were conducted with interaction terms between time (2019 or 2020) and job insecurity, fear of COVID-19, changes at work during COVID-19 and quality of management during COVID-19. Analyses were carried out using SAS version 9.4.

## Results

### Characteristics of respondents

As shown in table 1, high job insecurity was most frequent among employees from the hospitality sectors (13%), and least frequent in public administration and education (5%). We found the highest percentage of employees discharged with wage compensation in the hospitality industry (27%) followed by trade and transport (17%), whereas working from home was most frequent in public administration and education (77%) and other liberal professions (64%), and least frequent in health and social care (25%).

**Table 1:** Characteristics of responders, overall and stratified by industry

	Industries from the primary and secondary sector	Trade and transport	Hospitality	Other liberal professions	Public administration and education	Health- and social care	All industries
*N*	345	299	133	349	410	459	1995
Sex							
Male	235 (68)	159 (53)	34 (26)	184 (53)	137 (33)	67 (15)	816 (41)
Female	110 (32)	140 (47)	99 (74)	165 (47)	273 (67)	392 (85)	1179 (59)
Age							
Mean (SD)	48.8 (10.8)	46.1 (12.9)	43.4 (14.6)	47.2 (12.1)	47.9 (11.2)	47.7 (10.7)	47.3 (11.8)
15–29	20 (6)	39 (13)	31 (23)	43 (12)	34 (8)	32 (7)	199 (10)
30–44	94 (27)	69 (23)	31 (23)	87 (25)	114 (28)	124 (27)	519 (26)
45–59	181 (52)	158 (53)	50 (38)	166 (48)	199 (49)	242 (53)	996 (50)
60+	50 (14)	33 (11)	21 (16)	53 (15)	63 (15)	61 (13)	281 (14)
Educational level (register based)							
Primary school	50 (14)	53 (18)	32 (24)	40 (11)	21 (5)	29 (6)	225 (11)
Vocational and short-term higher education	195 (57)	172 (58)	47 (35)	134 (38)	94 (23)	143 (31)	785 (39)
Medium and long higher education	100 (29)	74 (25)	54 (41)	175 (50)	295 (72)	287 (63)	985 (49)
Fixed employment	331 (96)	272 (91)	108 (81)	331 (95)	384 (94)	426 (93)	1852 (93)
Workability, mean (SD)^a^	8.5 (1.8)	8.5 (1.8)	8.3 (1.8)	8.5 (1.7)	8.5 (1.7)	8.4 (1.8)	8.5 (1.8)
High job insecurity	30 (9)	28 (9)	17 (13)	34 (10)	20 (5)	28 (6)	157 (8)
High degree of fear of contracting COVID-19	119 (34)	90 (30)	48 (36)	100 (29)	111 (27)	163 (36)	631 (32)
High degree of fear of infecting others with COVID-19	163 (47)	145 (48)	73 (55)	157 (45)	179 (44)	258 (56)	975 (49)
Changes at work	241 (70)	217 (73)	100 (75)	271 (78)	358 (87)	401 (87)	1588 (80)
Discharged with wage compensation	36 (10)	52 (17)	36 (27)	23 (7)	55 (13)	62 (14)	264 (13)
9–12 weeks	6 (2)	15 (5)	16 (12)	7 (2)	13 (3)	8 (2)	65 (3)
Physical attendance at work	234 (65)	188 (63)	45 (34)	165 (47)	125 (30)	315 (69)	1062 (53)
9–12 weeks	132 (38)	108 (36)	20 (15)	66 (19)	36 (9)	169 (37)	546 (27)
Working from home	113 (33)	100 (33)	53 (40)	223 (64)	315 (77)	117 (25)	921 (46)
9–12 weeks	48 (14)	41 (14)	17 (13)	110 (32)	158 (39)	35 (8)	431 (22)

Values are number of respondents (percentages) unless stated otherwise.

a Respondents were asked to assess their workability on a scale from 0 (worst) to 10 (best).

About one-third (32%) of the respondents reported a high degree of fear of contracting COVID-19, and about half (49%) reported a high degree fear of infecting others with COVID-19. A high degree of fear of infecting others was most frequent among employees from health- and social care (56%) and the hospitality industry (55%) and least frequent in public administration and education (44%) and other liberal professions (45%). A high degree of fear of contracting COVID-19 was also most frequent in health- and social care (36%) and the hospitality industry (36%) followed by employees in industries from the primary and secondary sector (34%), and least frequent in public administration and education (27%) and other liberal professions (29%).

### Mental wellbeing by industry

A decrease in mean SWEMWBS was seen across all industries from 25.5 in 2019 to 24.4 in 2020 (*P* < 0.01) (table 2). There was no significant difference between industries [*P*(for interaction) = 0.064), although, compared to employees from public administration and education, the decrease in mental wellbeing was less pronounced among employees working in industries from the primary and secondary sector [estimate for interaction 0.7 (95% CI, 0.1; 1.3)]. No difference was found between the other industries (table 2).

**Table 2:** Mean SWEMWBS scores at time 2019 and 2020 by sector and generalized linear regression model of interaction between time and industry

Sector		SWEMWBS 2019	SWEMWBS 2020		
	*N*	Mean (95% CI)	Mean (95% CI)	Estimate^a^	*P*-value^b^
All industries	1995	25.5 (25.2; 25.7)	24.4 (24.2; 24.6)		<0.001
Industries from the primary and secondary sector	345	25.0 (24.5; 25.5)	24.5 (24.1; 25.0)	0.7 (0.1; 1.3)	0.064
Trade and transport	299	25.7 (25.2; 26.2)	24.2 (23.7; 24.6)	−0.3 (−1.0; 0.3)	
Hospitality	133	24.8 (24.0; 25.6)	24.0 (23.2; 24.7)	0.3 (−0.6; 1.3)	
Other liberal professions	349	25.3 (24.8; 25.8)	24.1 (23.7; 24.5)	−0.1 (−0.7; 0.5)	
Public administration and education	410	25.9 (25.5; 26.3)	24.7 (24.3; 25.1)	Reference	
Health- and social care	459	25.5 (25.1; 25.9)	24.4 (24.1; 24.8)	0.1 (−0.4; 0.6)	

a Interaction estimates adjusted for sex and age. The estimate is the additional effect of the industry category compared to the reference group (public administration and education).

b The *P*-values for the analysis of all industries is the influence of time (2019 vs. 2020). For the six industries, the *P*-values are an overall type-3 test of interaction between time (2019 and 2020) and industries (df = 5).

### Risk- and protective factors for mental wellbeing during COVID-19

We found no difference in changes in mental wellbeing among employees who experienced high job insecurity compared to those with low job insecurity and among employees experiencing a high degree of fear of contracting COVID-19 or a high degree of fear of infecting others (table 3). Moreover, no difference was found among employees discharged with wage compensation or those who attended work physically. However, employees working from home experienced a stronger decrease in mental wellbeing compared to those who were not [estimate for interaction −0.5 (−0.9; −0.2)]. Finally, the analyses showed that poor management quality is related to a stronger decrease in mental wellbeing. Thus, employees who were unsatisfied with their management’s communication about changes experienced a higher decrease in mental wellbeing compared to those who were satisfied [estimate for interaction −0.7 (−1.1; −0.3)]. Finally, employees who were unsatisfied with their managers’ involvement in changes [*P*(for interaction) = 0.002] and management of changes [*P*(for interaction) < 0.001] experienced a higher decrease in mental wellbeing.

**Table 3:** Mean SWEMWEBS scores at time 2019 and 2020 and differences by job insecurity, fear of COVID-19, changes at work during COVID-19 and quality of management during COVID-19. Linear regression model with repeated statement

		SWEMWBS 2019	SWEMWBS 2020		
	*N*	Mean (95% CI)	Mean (95% CI)	Estimate^a^	*P*-value^b^
Job insecurity					
High job insecurity	157	23.4 (22.7; 24.2)	22.1 (21.6; 22.6)	−0.3 (−0.9; 0.4)	0.41
Low job insecurity	1838	25.6 (25.4; 25.8)	24.6 (24.4; 24.8)	Reference	
Fear of COVID-19					
High degree of fear of contracting COVID-19	631	25.1 (24.7; 25.4)	23.8 (23.5; 24.1)	−0.3 (−0.7; 0.2)	0.25
Low fear	1364	25.6 (25.4; 25.9)	24.6 (24.4; 24.8)	Reference	
High degree of fear of infecting others with COVID-19	975	25.2 (24.9; 25.5)	24.2 (24.0; 24.5)	0.2 (−0.1; 0.6)	0.21
Low fear	1020	25.7 (25.5; 26.0)	24.5 (24.3; 24.8)	Reference	
Changes at work during COVID-19					
Changes during lock down	1588	25.4 (25.2; 25.6)	24.3 (24.1; 24.5)	−0.1 (−0.6; 0.4)	0.64
No changes	407	25.7 (25.2; 26.2)	24.7 (24.3; 25.1)		
Discharged with wage	264	24.8 (24.2; 25.3)	24.0 (23.5; 24.5)	0.4 (−0.2; 0.9)	0.16
Not discharged	1731	25.6 (25.3; 25.8)	24.4 (24.2; 24.6)	Reference	
Physically attended work	1062	25.4 (25.1; 25.7)	24.5 (24.2; 24.7)	0.4 (−0.0; 0.7)	0.066
No physical attendance	933	25.5 (25.2; 25.8)	24.3 (24.0; 24.5)	Reference	
Worked from home	921	25.9 (25.6; 26.2)	24.6 (24.3; 24.8)	−0.5 (−0.9; −0.2)	0.005
Did not work from home	1074	25.0 (24.8; 25.3)	24.2 (24.0; 24.5)	Reference	
Quality of management during COVID-19					
Unsatisfied with level of information	508	24.7 (24.4; 25.1)	23.1 (22.8; 23.5)	−0.7 (−1.1; −0.3)	<0.001
Satisfied	1487	25.7 (25.5; 25.9)	24.8 (24.6; 25.0)	Reference	
Unsatisfied with level of involvement	976	25.1 (24.9; 25.4)	23.8 (23.5; 24.0)	−0.6 (−1.0; −0.2)	0.002
Satisfied	1019	25.7 (25.5; 26.0)	25.0 (24.7; 25.2)	Reference	
Unsatisfied with management of changes	664	25.0 (24.7; 25.4)	23.5 (23.2; 23.8)	−0.7 (−1.1; −0.4)	<0.001
Satisfied	1331	25.7 (25.4; 25.9)	24.8 (24.6; 25.0)	Reference	

a Linear regression adjusted for sex and age. The estimates are the interaction estimate, i.e. the additional effect of the stratifying variable in addition to time and the variable.

b The *P*-values are a test of interaction between time (2019 and 2020) and the stratifying variable.

## Discussion

In this study, we had unique survey data from a representative sample of the Danish population, and unlike most other studies, we measured mental wellbeing just before the onset of the pandemic and followed the same population during the COVID-19 pandemic.^18^ We found a decrease in mean SWEMWBS across all industries from 25.5 in 2019 to 24.4 in 2020. The mean decrease corresponds to a minimally important level of change.^22^ The results do not support our hypothesis that the COVID-19 pandemic has had a more negative effect on mental wellbeing among employees in the hospitality industry and health/social care industry compared to other industries. Several explanations may exist. First, the pandemic did not hit Denmark as hard as other European countries, such as Italy,^23^ and although unemployment increased by 0.8% from 2019 to 2020, job insecurity was lower in Denmark compared to other EU countries.^3^ If people believe that they can easily find a new job, job insecurity may not constitute a major resource threat. Moreover, the cumulative number of confirmed deaths per million people in Denmark is 448 (8 September 2021). This is lower compared to the USA (1960) and most other European countries, including Sweden with 1447 confirmed deaths per million people, but somewhat higher than Norway (151) and Finland (187).^24^ Danish employees in the hospitality industry and health/social care industry may have been less exposed to the negative consequences of the pandemic compared to employees in other countries. Results may therefore not be generalizable to other countries that have experienced a much higher burden on the health sector and economic backlash in the hospitality sector.

We found that employees working from home experienced a greater decline in mental health than those who continued going to their workplace, and that working from home was most prevalent in public administration, education and other liberal industries. Our findings show that working from home is negatively related to mental wellbeing compared to going to work physically, it may not apply directly to a post-pandemic scenario. During the pandemic many employees did not voluntarily work from home, and the workplaces were not prepared for this major change. Moreover, many people were isolated from friends and families and not able to pursue sport- and leisure time activities.

Research on working from home during the pandemic remains scarce and has produced somewhat conflicting results on the mental and physical impact. Xiao *et al*.^25^ found that office workers, who transitioned to working from home during the pandemic, reported a decline in mental health, and that decreased physical activity, increased junk food intake, lack of communication with co-workers and having a toddler at home predicted poor physical and mental health. Moreover, one Swedish study among office workers found that working from home was associated with longer duration of sleep compared to days working at the office.^26^ In terms of physical activity and sedentary behaviour, the researchers found no change in sedentary, standing and moving behaviours, which they assessed objectively using accelerometers.^26^ In contrast, a study from the USA, encompassing 2303 US adults, found that switching to working from home during the COVID-19 pandemic was associated with greater time spent sitting and viewing screens.^27^

This study also contributes with knowledge about the importance of management in a time of considerable workplace changes. Although all employees experienced a decrease in mental wellbeing, the decrease was less pronounced among employees who received adequate information and who felt that managers involved them in decision making. This finding is in line with other studies and COR Theory. For instance, previous studies about successful measures to manage psychological distress have found that clear communication and both practical and psychological support were associated with reduced morbidity in clinicians working with novel outbreaks.^28^^,^^29^ Similarly, a study from the USA found that poor family-supportive behaviours by supervisors were associated with more negative mental health outcomes among staff working at a university and its medical centre.^30^ Management quality is a factor amendable to change, and previous research shows that training encompassing reflective and interactive parts in a group setting over several days seem to be the most promising strategy to address mental health in healthcare employees.^31^ Finally, in a time characterized by social distancing and other pandemic-related conditions, digital interventions to improve mental health at the workplace level and among the public is becoming increasingly important.^32^

Although the COVID-19 pandemic constitutes an unprecedented crisis, workplaces will have to adapt to rapid changes in technology, climate change, financial downturns etc. Moreover, working from home is now more widespread than before. Thus, learning from the pandemic will be valuable for preparing for future crises. Our study highlights the importance of prioritizing mental health promotion and to pay particularly attention to the effects of working from home. However, more research is needed to assess if mental wellbeing will return to pre-COVID levels to fully assess the consequences, and more research focusing on workplace resources (e.g. management quality and flexible working arrangements) will be important to plan mental health promoting efforts.

### Strengths and limitations

It is a major strength that the study is based on a representative sample with repeated measures of mental wellbeing, which we measured just before the outbreak of the pandemic in Europe. This allows us to take account of any pre-pandemic suboptimal mental wellbeing. It is also a strength that we collected data during autumn both years limiting the seasonal variations in mental wellbeing. However, when interpreting results, it is important to consider the timing of the study. We measured mental wellbeing after a summer characterized by low infection rates and a somewhat normalized situation. Schools and restaurants had reopened, and many employees had returned physically to their work, at least some of the time.

A weakness of the study is the rather low response proportion (2019: 47%; 2020: 50%). While the use of register-based data to assess which industry, respondents were employed in is a strength, it is a limitation that some of the categories were rather broad and heterogeneous. Thus, we were not able to look at health care and social work separately. Moreover, the tourism industry has also experienced marked economic difficulties in 2020,^2^ but we were not able to separate the tourism industry from other liberal professions. While we used validated measures to assess mental wellbeing and quality of management, the questions pertaining to fear of COVID-19 were constructed specifically for this study and was not based on a validated questionnaire, such as the newly published Fear-of-COVID-19 scale.^33^

## Funding

This work was supported by the Velliv Foreningen (20-0438).

*Conflicts of interest*: None declared.

## Data Availability

Data are linked to administrative registers and can only be accessed through affiliation with University of Southern Denmark. The authors welcome any contacts regarding collaboration. The questionnaires (in Danish) are accessible at https://www.sdu.dk/da/sif/forskning/projekter/betydningen_af_covid_19_krisen. Mental wellbeing declined among employees in all industries during the first COVID-19 wave in Denmark in 2020. Employees from health- and social care and the hospital industry were not more negatively affected than employees from other industries. Employees working from home experienced a stronger decrease in mental wellbeing compared to those who attended work physically. Poor management quality was related to a stronger decrease in mental wellbeing. Managers play a key role in mitigating the negative consequences of the pandemic by ensuring adequate information and involvement of employees.
